# Notes of a Rattle-Snake Bite Admitted into the Ill. Gen. Hospital

**Published:** 1852-01

**Authors:** John E. McGirr

**Affiliations:** One of the Surgeons to the Hospital, and Prof. of Chemistry and Physiology in the University of the Lake


### ARTICLE II.

*Notes of a Rattle-Snake Bite admitted into the Illinois General Hospital of the Lakes.* By JOHN E. MCGIRR, M.D., L. L. D., one of the Surgeons to the Hospital, and Prof. of Chemistry and Physiology in the University of the Lake.

Edward Donelly, laborer, was brought from the country twelve miles distant, to-day, August 13th, into the Hospital.

*History.*—He was bitten by a rattle-snake on the second phalanx of the index finger, right hand, yesterday, 12th inst., about 11 o'clock, A. M., while engaged in binding oats in the harvest field. Immediately after being bitten he put the finger into his mouth with the expectation of being able to suck out the poison. Donelly killed the snake, which had ten rattles upon it, and cutting it in two, he applied the cut surface of the tail end to the part bitten and walked with it thus applied, about a mile, to the first house. Here he had spts. ammonia, indigo, &c., applied to it. He then became insensible and the first thing he remembers after this was vomiting blood. At the same time that he vomited blood, the bitten part began to bleed; also, an old ulcer on the left leg, which had been healed except a space about the size of a dime. The blood vomited came from the mouth; he had swallowed it. The mouth, finger, and leg bled simultaneously and continuously.

Violent pain soon set in all over his body; this continued until about one o'clock at night when he got about two hour's sleep. The pain then returned and continued until next day, when dizziness, faintness, and partial loss of sight, occurred. The bleeding had never ceased, and, when he was brought into the Hospital, all the clothes about him were saturated with blood.

*Present Appearance.*—The finger livid, swollen, and streaming blood from the bitten part, around which the cuticle had separated, as if blistered. The right hand and arm were very much swollen. Spots of ecchymosis varying from the size of a pea to that of a shilling, covered the whole body; some of these were raised and hard to the touch. Patches of ecchymosis were on the arm near the axilla. Pulse full and quick. Tongue has a whitish fur, thick along the centre. Cadaverous smell from the mouth. Respiration labored. Whole system evidently under the influence of the poison.

Physical character of the blood examined by Mr. Johnson, *Interne*, was as follows: Blood does not coagulate, color deeper than natural; under a microscope of 400 diameters, corpuscles sphericle, unnatural number of nuclei; not a trace of fibrine. Blood presents much the same appearance as healthy blood acted upon by acetic acid.

*Treatment.*—Professor Herrick, one of the Surgeons to the Hospital, kindly prescribed at once for the case in my absence. He ordered at one o'clock P. M., the following :

℞. Potass. Iodide gr. V. To be given every three hours.

℞. Chloride Sodium gr. X V, to alternate.

He directed mattico to be applied to the bleeding finger and to the bleeding ulcer. I came in time to apply the mattico. It was bandaged down to the bleeding surfaces ; the fingers, hand and arm were also bandaged, and acetas plumbi gr. IV, pulv. opii gr. I, directed to be given every hour should the bleeding recur.

At seven o'clock P. M., the bleeding had not recurred from either finger or ulcer. Prescription of Professor Herrick continued.

14th. Did well till about one o'clock to-day, when the bleeding began again from the finger and ulcer and continued till two o'clock P. M. One powder of acetate of lead and opium, as above, given. Bleeding returned at five P. M. The bleeding surfaces dressed and fresh mattico applied which arrested the discharge. The same fœtid, or cadaverous smell of the mouth continues, root of the tongue stiff and sore, occasional starts of pain the in finger, pulse 84, hard ; violent pain in the hand and arm during the evening.

℞. Carb ammonia gr. iij ; camphor gr. iij, every two hours.

16th. About 4 o'clock this morning the breathing became somewhat oppressed. Pulse, 96. Ecchymosis on the front part of the arm extends in one continuous patch to the axilla. The cuticle on the back of the finger all puckered as if seared. It is raised and hardened. The back of the hand is distended with fluid. The examination, of the blood under the microscope shows the gradual restoration of the fibrine. If astringents be given to arrest the discharge of blood from the mucous membrane of the mouth, the finger bleeds---one or other bleeds slightly without ceasing.

℞. ol terebinthina gtt XXV in emulsion every six hours until the bleeding is arrested.

The spots of ecchymosis, before spoken of, begin to fade from the centre towards the circumference, restoring the natural color. The blood drawn the first day of the patients admission remains in the vial dark and liquid with the same cadaverous smell precisely as that given from the mouth, which arose from the blood constantly discharing from it.

17th. Much improved. The bleeding from the mouth stopped altogether, and it is very slight from the finger. Ecchymosis growing fainter, although a large patch has appeared upon the back of the left arm above the elbow. Continue ol. terebinthina.

18th. Not quite so well to-day ; last night had a very severe chill, followed by fever and sweating. Continue former prescription and add

℞. Quinia gr. V.

19th. ℞. Quinia sulph. gr. iij. Carb. ferri. gr. iij.

One powder to be given every four hours with wine. Has had no return of chilliness ; bleeding altogether arrested.

Nothing worthy of note further occurred to arrest speedy recovery. The first phalanx sloughed away and considerable sloughing took place on the front of the second, which exposed a portion of the bone. This become carious and was removed when the finger healed up entirely.

On the 26th of August the man left the Hospital.

